# Aseptic and Alopecic Nodule of the Scalp in a Young Female With Alopecia Areata

**DOI:** 10.1155/crdm/1835329

**Published:** 2026-04-07

**Authors:** Kara Turner, Arielle Carolina Mora Hurtado, Brittney DeClerck, Nada Elbuluk

**Affiliations:** ^1^ Albert Einstein College of Medicine, Bronx, New York, USA, yu.edu; ^2^ University of Wisconsin School of Medicine and Public Health, Madison, Wisconsin, USA, uwm.edu; ^3^ Department of Dermatology, Keck School of Medicine of the University of Southern California, Los Angeles, California, USA, usc.edu

**Keywords:** alopecia areata, aseptic and alopecic nodules of the scalp, pseudocyst of the scalp

## Abstract

Aseptic and alopecic nodules of the scalp (AANS) is a rare, likely underrecognized, nonscarring alopecia characterized by one or more alopecic nodules without evidence of microbial infection. AANS has a favorable prognosis and often responds well to treatments such as doxycycline, intralesional steroids, or drainage. We present a case of AANS in a young female with a history of biopsy‐proven alopecia areata that achieved full resolution without recurrence with intralesional steroids. Recognition of this rare condition, including in the setting of other established alopecia diagnoses, may avoid a missed diagnosis as well as unnecessary surgery.

## 1. Introduction

Aseptic and alopecic nodules of the scalp (AANS) is a rare, inflammatory, nonscarring alopecia that presents with one or more alopecic nodules in the absence of microbial growth [1–4]. While the etiology is unclear, it is postulated that follicular occlusion may contribute to its pathogenesis [1, 2, 5, 6]. The condition predominantly affects young males [1]. Nodules can be soft, firm, or fluctuant [1]. While pruritus or tenderness can occur, nodules are frequently asymptomatic [1, 4]. Histopathology often demonstrates a granulomatous infiltrate with lymphocytes, neutrophils, plasma cells, and histiocytes within the deep dermis [1]. AANS has a favorable prognosis, responding well to doxycycline, intralesional steroids, or drainage [4, 6]. We present a case of AANS in a patient with a history of alopecia areata treated with intralesional steroids that achieved full resolution. As AANS and alopecia areata both present with nonscarring alopecia, their overlapping clinical features warrant further diagnostic evaluation, including in patients with established alopecia diagnoses. This case illustrates the importance of thorough history‐taking and that patients may carry multiple distinct alopecia diagnoses.

## 2. Case Report

**FIGURE 1:**
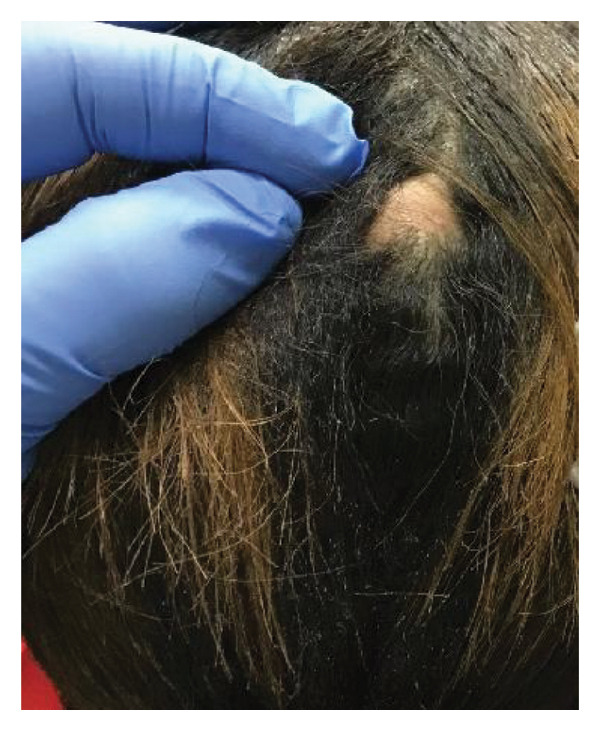
Single dome‐shaped skin‐colored nodule with overlying alopecia on the vertex of the scalp.

A 22‐year‐old female with a history of biopsy‐proven alopecia areata presented with several months of a tender, pink, boggy nodule with overlying nonscarring alopecia on the vertex of the scalp (Figure 1). Alopecia areata had not been active at the time of symptom onset. The patient had no known personal of family history of follicular occlusion disorders, including acne, though she presented with few axillary cysts at a later visit. Trichoscopy demonstrated yellow dots in the center of the lesion and fine vellus hairs at the periphery. There were no broken hair shafts visible. The nodule was injected with intralesional steroid (ILK). One month later, the nodule was nontender though still boggy and associated with alopecia. The nodule underwent a second ILK injection with no significant change. Ultrasound was obtained which was unremarkable.

Persistence of the nodule at subsequent visits led to evaluation by biopsy. When punctured, the lesion drained blood‐tinged fluid. Punch biopsy revealed alopecia with a focus of granulation tissue with lymphocytes, histiocytes, neutrophils, and plasma cells within the deep dermis (Figure 2). The bacterial and fungal cultures of the nodule were negative. Based on the clinical and histopathologic findings, a diagnosis of AANS was made.

FIGURE 2Skin biopsy showing (a) horizontal section of superficial dermis showing nonscarring, pauci‐inflammatory alopecia with some miniaturization of hairs (hematoxylin and eosin; magnification: ×  10) and (b) deep dermis with a focus of granulation tissue and reactive changes (hematoxylin and eosin; magnification: ×  20).

**Figure:**
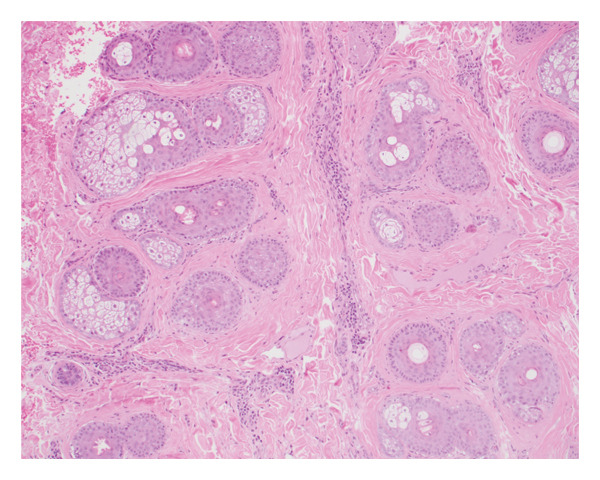
(a)

**Figure:**
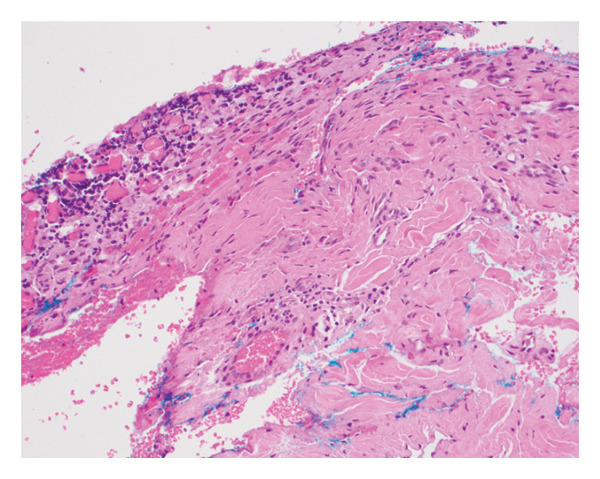
(b)

**FIGURE 3:**
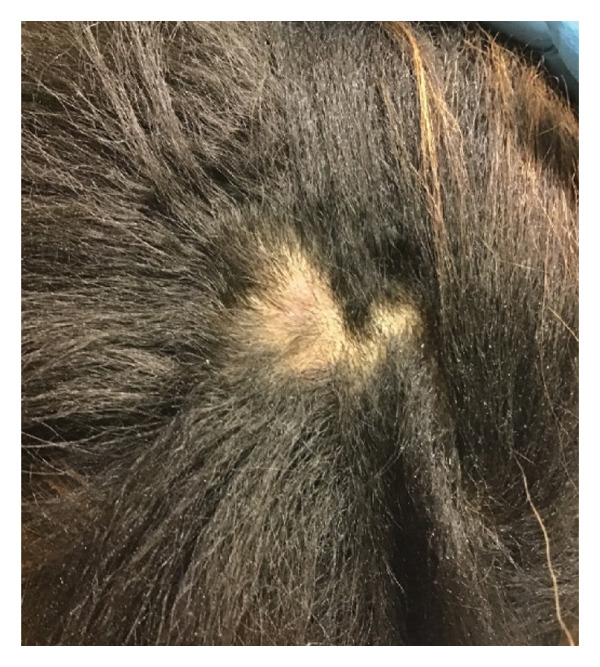
Posttreatment decrease in nodule size with interval hair regrowth.

Treatment consisted of ILK monthly for two additional months. At a 3‐month follow‐up visit, there was a full resolution of symptoms with a decrease in nodule size and regrowth of hair (Figure 3). No recurrence was reported.

## 3. Discussion

AANS is a rare nonscarring alopecia though a variety of case reports indicate that the condition is likely underrecognized and may be more common [1]. The term “AANS” was first coined by Abdennader and Reygagne in 2009 [7]. However, a similar condition known as pseudocyst of the scalp (PCS) was first described by Iwata et al. [8] in 1992 in a series of case reports. Currently, it is unclear whether these two entities represent a spectrum of the same condition or two distinct diseases [1]. The cases reported by Iwata et al. described PCS as presenting with alopecic nodules between the top and the forehead of the scalp. When punctured, they drained citrine‐colored or blood‐tinged liquid with histopathology showing a pseudocyst‐like architecture with granulomatous infiltration [1, 3, 8]. The literature describing AANS has reported alopecic nodules in the vertex and upper part of the occiput [1, 7]. When punctured, the drainage from these nodules was sometimes purulent. Histopathology showed a nonspecific mixed infiltration and usually a granuloma in the deep dermis, and pseudocystic architecture was not always present [1, 3]. Whether the distinct histopathological findings may be attributed to differences in hair type, severity, and stage of disease or two separate disease processes altogether remains to be elucidated. In this case, the lesion presented on the scalp vertex, and there was no pseudocystic architecture detected on histopathology, aligning more closely with AANS.

AANS primarily affects young males, aged 20 to 30 years. The youngest patient observed was a 7‐year‐old boy, and the oldest was a 72‐year‐old woman [5, 9]. The etiology of AANS remains to be fully elucidated, but follicular occlusion is thought to play a role in its pathogenesis. An immune response to a form of deep folliculitis may induce a granulomatous inflammatory reaction against the hair follicle, triggering nodule or pseudocyst formation [7]. Some hypothesize that the inflammatory reaction may also be due to follicular alteration, a foreign body, or another unknown factor [3]. Further studies are needed to clarify the pathogenesis of AANS.

Clinically, the condition is characterized by one or more dome‐shaped nodules that may be soft, firm, or fluctuant associated with alopecia and normal surrounding scalp [1–4, 7]. Nodules are often asymptomatic but may be mildly painful or pruritic. Bacterial and fungal cultures obtained from AANS are always sterile [7]. Differential diagnoses are several and may include dissecting cellulitis, pilar cyst, and metastatic nodules [1–4, 6]. Trichoscopy may reveal black and yellow dots, fine vellus hair, and broken hair shafts, and it can be a useful diagnostic tool, particularly in distinguishing AANS from dissecting cellulitis, which characteristically reveals scarring and large three‐dimensional yellow dots imposed over dystrophic hairs [2]. Ultrasonography is another noninvasive technique that may be helpful in diagnosis. Ultrasonography of AANS demonstrates a well‐defined subcutaneous hypoechoic nodule without areas of fluid collections or abscesses connecting hypoechoic fistulous tracts reaching the hair bulb [2, 3].

Overall, AANS has a favorable prognosis. Resolution of AANS may occur spontaneously or after drainage or aspiration [1]. Intralesional corticosteroids, doxycycline, and nonsteroidal topical treatments have also been reported as successful treatments [1, 3]. Typically, surgical excision is not necessary, and recognition of AANS may avoid unnecessary surgical treatments [2, 4]. Further research is needed to determine the optimal treatment for AANS.

To our knowledge, no other cases of AANS have been reported concurrently in patients with alopecia areata. This case illustrates a unique presentation of a rare and underrecognized condition that dermatologists should be aware of including in the setting of other alopecia diagnoses.

## Author Contributions

Kara Turner and Arielle Carolina Mora Hurtado contributed to writing the manuscript, and Brittney DeClerck and Nada Elbuluk supervised the work.

## Funding

This study was not supported by any sponsor or funder.

## Disclosure

All authors have read and approved the final manuscript.

## Ethics Statement

This retrospective review of patient data did not require ethical approval in accordance with local/national guidelines.

## Consent

Written informed consent was obtained from the patient for publication of this case report and any accompanying images.

## Conflicts of Interest

Dr. Nada Elbuluk has served as a consultant, advisory board member, and/or speaker for Avita, Scientis, Incyte, VisualDx, La Roche Posay, Beiersdorf, Unilever, Eli Lilly, Galderma, Pfizer, L’Oreal, McGraw Hill, Dior, Medscape, AbbVie, Takeda, Sanofi, Janssen, and Canfield22. She has received royalties from McGraw‐Hill. She has stock options in VisualDx. The remaining authors declare no conflicts of interest.

## Data Availability

The data that support the findings of this study are not publicly available due to privacy reasons but are available from the corresponding author upon reasonable request.
